# Run and hide: visual performance in a brittle star

**DOI:** 10.1242/jeb.236653

**Published:** 2021-06-08

**Authors:** Lauren Sumner-Rooney, John D. Kirwan, Carsten Lüter, Esther Ullrich-Lüter

**Affiliations:** 1 Oxford University Museum of Natural History, University of Oxford, Parks Road, Oxford OX1 3PW, UK; 2 Stazione Zoologica Anton Dohrn, Via Francesco Caracciolo, 333, 80122 Naples, Italy; 3 Museum für Naturkunde, Leibniz Institute for Evolution and Biodiversity, Invalidenstrasse 43, 10115 Berlin, Germany

**Keywords:** Echinoderms, Extraocular vision, Ophiuroids, Vision

## Abstract

Spatial vision was recently reported in a brittle star, *Ophiomastix wendtii*, which lacks discrete eyes, but little is known about its visual ecology. Our aim was to better characterize the vision and visual ecology of this unusual visual system. We tested animal orientation relative to vertical bar stimuli at a range of angular widths and contrasts, to identify limits of angular and contrast detection. We also presented dynamic shadow stimuli, either looming towards or passing the animal overhead, to test for potential defensive responses. Finally, we presented animals lacking a single arm with a vertical bar stimulus known to elicit a response in intact animals. We found that *O. wendtii* orients to large (≥50 deg), high-contrast vertical bar stimuli, consistent with a shelter-seeking role and with photoreceptor acceptance angles estimated from morphology. We calculate poor optical sensitivity for individual photoreceptors, and predict dramatic oversampling for photoreceptor arrays. We also report responses to dark stimuli moving against a bright background – this is the first report of responses to moving stimuli in brittle stars and suggests additional defensive uses for vision in echinoderms. Finally, we found that animals missing a single arm orient less well to static stimuli, which requires further investigation.

## Abstract (precis)

**Summary:** Brittle star responses to static visual stimuli of varying size and contrast show that visually guided habitat selection requires large, high-contrast stimuli. Responses to the appearance of overhead shadows reveal additional uses for photoreception in defensive behaviours.

## INTRODUCTION

Light is a critical biological cue, and detecting it can play a role in habitat selection, defensive behaviour, ultraviolet (UV) protection and biological rhythms. Image-forming vision can facilitate more complex behaviours including navigation, communication and pursuit (Nilsson, 2013). Eyes – discrete sensory organs that facilitate vision – have evolved dozens of times among animals and exhibit a stunning diversity of forms (Land and Nilsson, 2012; von Salvini-Plawen and Mayr, 1977). Many animals also detect light using extraocular photoreceptors, in the brain, skin, in intrinsically light-sensitive neurons, or elsewhere (e.g. Battelle, 2016; Kingston and Cronin, 2016; Ramirez et al., 2011; Tong et al., 2009). Until recently, it was considered that these extraocular photoreceptors did not contribute to vision or visual processes (reviewed in Cronin and Johnsen, 2016).

Recent experiments demonstrate extraocular photoreceptors mediating vision in echinoderms (Blevins and Johnsen, 2004; Kirwan et al., 2018; Sumner-Rooney et al., 2020; Yerramilli and Johnsen, 2010). In both sea urchins (Ullrich-Lüter et al., 2011) and brittle stars (Delroisse et al., 2014; Johnsen, 1997; Sumner-Rooney et al., 2018), putative photoreceptors are spread over the body surface, and in select species these appear to mediate responses to visual stimuli, undetectable by non-visual photoreception (Kirwan et al., 2018; Sumner-Rooney et al., 2020).

The occurrence of extraocular vision in at least two classes of echinoderms opens up a host of new questions and potential research avenues concerning their function, limitations, signal integration and evolution. Our fundamental understanding of vision has been built on centuries of work on animal eyes. Distributed visual systems, including those of ark clams and fan worms (Nilsson, 1994), break from many of the conventions of paired eyes, but still retain discrete visual units. Extraocular systems, however, cannot be easily described optically because photoreceptors are not arranged in adjacent rows within a discrete structure, making properties such as acceptance angle more difficult to estimate. These animals therefore represent a challenge for visual biologists. With a better understanding of extraocular visual systems and their capabilities, we may also find new resources and inspiration for biomimetic sensor technology (e.g. Aizenberg and Hendler, 2004; Mao et al., 2013; Watanabe et al., 2011; Yang and Aizenberg, 2005).

The brittle star *Ophiomastix wendtii* (Müller and Troschel 1842) (formerly *Ophiocoma wendtii*, according to O'Hara et al., 2019) orients to isoreflectant stimuli. These reflect the same amount of light, in total, as the background against which they are presented, and are therefore detectable only via object-resolving vision (Sumner-Rooney et al., 2020). *Ophiomastix wendtii* inhabits densely populated reef rubble in the Caribbean and Gulf of Mexico, where predation pressure is high. Parrotfish, wrasse and mojarra are all reported to feed on members of the genus, with the greatest threat from *Sparisoma* and *Halichoeres* spp. (Hendler, 1984). These are large, fast-moving diurnal species that are present in high numbers, and particularly parrotfish and mojarra may consume whole animals (Hendler, 1984). Orientation to these static stimuli may reflect motivation to seek shelter in bright, heterogenous reef rubble habitats, and that animals are attracted to regions of local contrast that might indicate structural heterogeneity and therefore potential refuge from predation (Sides and Woodley, 1985).

Clarifying the visual acuity exhibited by *Ophiomastix* would shed light on both visual function and visual ecology in relation to this behaviour; for example, Sides and Woodley (1985) suggested that *O*. *wendtii* maintained sympatry with other ophiocomids by selecting larger crevices in which to shelter. This could reflect a limit on acuity or a selective preference. Moreover, high-contrast signals (as used by Sumner-Rooney et al., 2020) are uncommon in oceanic water, and investigating contrast sensitivity at detectable spatial frequencies (once identified) helps infer how performance and visual ecology interrelate. Although the habitat of *O*. *wendtii* is shallow and bright, we might expect animals to respond to lower contrast signals more representative of natural scenes if this behaviour is biologically relevant.

Other uses for low-resolution vision may exist, besides habitat selection. To date, only static stimuli have been tested in *Ophiomastix*, but their natural visual environment is busy and dynamic. Responses to moving stimuli might reveal additional or alternative roles of vision in predator detection. In *Diadema*, both the sudden and looming appearance of dark stimuli against a bright background elicited a characteristic defensive spine-pointing response. In other distributed visual systems, including chiton shell eyes, ark clam compound eyes and fanworm radiolar eyes, predator detection is the core task of vision (Bok et al., 2019; Nilsson, 1994; Speiser et al., 2011). Other than arm autotomy, defensive or harm-reductive behaviours are not extensively documented in brittle stars. Hendler (1984) reported that arm spines would raise and lock into place and that animals produce mucus when they are mechanically disturbed. The nocturnal activity patterns and proposed shelter-seeking of *O*. *wendtii* may contribute to their avoidance of predation, but given their vulnerability to fast-moving visual predators during the day, we might expect them to respond to moving shadows.

How animals might integrate information from thousands of dispersed photoreceptors remains uncertain. Sumner-Rooney et al. (2020) highlighted a distinction in locomotion between sighted *O. wendtii* and a closely related but apparently non-sighted species *Ophiocomella pumila*, which they proposed was a possible clue to signal integration. Leading with a single arm (‘rowing’; Astley, 2012) appears to be the dominant locomotory mode in most ophiuroid species studied to date (Arshavskii et al., 1976; Astley, 2012; Clark et al., 2018; Glaser, 1907; Wakita et al., 2020). ‘Reverse rowing’ (Astley, 2012), with two leading arms, is also widely reported but seems to be less common. While *O. pumila* almost always moved by rowing, *O. wendtii* used reverse rowing in the majority of experiments (Sumner-Rooney et al., 2020). These authors suggested that leading with two outstretched arms could indicate signal comparison between them in *O. wendtii*; the complex and variable body shape and relatively rapid movement of *O. wendtii* probably presents significant additional challenges for extraocular vision in comparison with the fixed spherical shape of sea urchins. Comparing pooled photoreceptor signals between adjacent arms could be a much simpler method of contrast detection than integrating spatial signals within arms or across the whole body. Previous authors have suggested that each arm acts as a semi-autonomous system (reviewed in Yoshida, 1966), and Stubbs (1983) demonstrated that electrical responses to the illumination of one arm can be detected in the radial nerve cord of another. If communication between adjacent arms was used during stimulus detection and approach, arm damage or loss could impede this ability. Although brittle stars readily autotomise arm portions or entire arms during defensive behaviours, previous experiments have been restricted to intact animals. We therefore repeated one of the experiments of Sumner-Rooney et al. (2020) using animals missing a single arm to study the effects of arm loss on orientation responses.

We aim to better characterise the visual performance of *O. wendtii*, identifying fundamental thresholds to visual behaviour and testing responses to established dynamic stimuli. In determining these thresholds, we hope to gain a better understanding of the visual ecology, and ultimately, the evolution of this unusual visual system.

## MATERIALS AND METHODS

### Specimens

Adult *Ophiomastix wendtii* were collected in May–June 2017 and June–July 2019 at Punta Hospital, Isla Solarte, Panama (9°20′00.7″N, 82°13′03.6″W; 0–3 m), and housed in open-air aquaria at the Smithsonian Tropical Research Institute (STRI) in Bocas del Toro, Panama (MiAMBIENTE research permits SE/A-35-17 and SE/A-48-19). Animals were given 3 days to recover from collection before experiments. Those that autotomised arms were excluded from experiments unless otherwise stated. Animals were exposed to each stimulus only once and were tested up to twice per day. Animals collected in 2017 measured a mean (±s.d.) of 105.8±21.6 mm from the centre of the disc to the tip of the longest arm. Animals collected in 2019 measured 107.4±17.3 mm. Sex of the animals was unknown.

### Orientation experiments

**Fig. 1.**
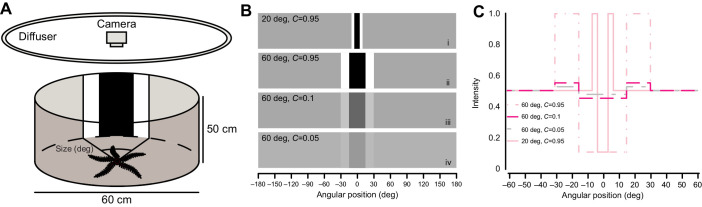
**Orientation experiments in *Ophiomastix wendtii*.** (A) Animals were placed at the centre of a circular arena with a vertical bar stimulus presented at one side and their movements were filmed from above. (B) Stimuli varied in angular width (i,ii) or in Michelson contrast (iii,iv). (C) Intensity profiles of the four example stimuli i–iv, demonstrating variation in width and amplitude.

Animals were placed at the centre of a circular arena (diameter 60 cm, depth 50 cm) filled with unfiltered seawater, and with a stimulus pattern presented on the inside wall on printed vinyl (Fig. 1A). Animals move linearly from the centre to the edge of the arena (Sumner-Rooney et al., 2020). We measured angular bearings of animal position (both absolute and relative to the centre of the stimulus) when the disc first touched the arena wall. The position of the stimulus was changed between every trial, being randomly selected each time from a list of eight positions at 45 deg increments (i.e. 0, 45, 90 deg, etc.). The arena floor and walls were cleaned, and seawater was replenished, between trials. Experiments were performed in natural daylight beneath a diffuser consisting of multiple layers of white cotton. Solar irradiance is monitored by the STRI physical monitoring programme using a Kipp & Zonen SP Lite2 pyranometer (sensitivity 400–1100 nm), with mean irradiance being measured every 15 min. Experiments were conducted between 10:00 h and 15:15 h to avoid spectral impacts of sunrise (06:10–06:15 h) and sunset (18:45–18:50 h). To ensure that the stimuli reflected proportionately, the reflectance of a series of test pieces (of printed vinyl with relevant grey values) was measured using an RPS900-R spectroradiometer (International Light, Peabody, MA, USA) in air. The spectroradiometer recorded the relative counts of light reflected by each of the test pieces and by a highly reflective white standard. Counts were measured across the visible range of wavelengths (300–700 nm, at 0.45 nm intervals). All stimulus contrasts are Michelson contrasts (*C*), given by:(1)
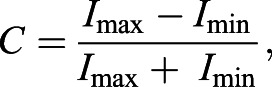
where *I*_max_ and *I*_min_ are the maximum and minimum values of the stimulus (darker and lighter vertical bars).

### Stimulus size

To find the resolution to which animals respond, subjects were presented with discrete bar stimuli: a central black target with white flanks half as wide, with a total angular width from 20 to 70 deg (in 10 deg increments) of the arena walls, which were elsewhere an intermediate grey. The reflectance of black and white areas within each stimulus counterbalanced one another, making stimuli isoreflectant to the background, as confirmed by reflectance measurements (Fig. 1B,C). These black and white stimuli had a Michelson contrast of 0.95 (due to reflectance in the dark region). Experiments using a stimulus of 50 deg width were performed in May–June 2017; all other experiments were performed using an identical set-up, animals of similar size (see ‘Specimens’ section above), and following identical procedures in June–July 2019.

### Contrast sensitivity

To assay whether *O. wendtii* detects low contrasts, two variants of the 60 deg centred vertical bar stimulus were added. These differed from the main stimulus in having proportionately reduced amplitude light and dark bars, with the Michelson contrast between the light and dark parts reduced from 0.95 to 0.1 and 0.05, respectively. The total stimuli (light and dark bars combined) were isoreflectant with the background of the arena (Fig. 1B,C).

### Arm loss

We tested 29 animals that autotomised a single arm (to below a quarter of adjacent arm length), as above, for orientation to a 60 deg stimulus of Michelson contrast 0.95. Animals were placed in the centre of the arena with the missing arm randomly oriented in relation to the stimulus centre. Initial body orientation, terminal body orientation and terminal bearing were recorded.

### Analysis

To analyse orientation data (bearings at the end of a trial), we used two approaches, either treating the data as circular, and distributed according to a von Mises distribution, or discretized: partitioned into success if they fell within a 60 deg sector centred on the stimulus midpoint, or failure otherwise.

The orientedness of the absolute (to true north) bearings combined across all treatments was compared against a range of orientation models using maximum likelihood estimates to test for a general directional bias using the Circ_MLE package in R (Fitak and Johnsen, 2017). All further analyses used bearings relative to the stimulus midpoint.

We assessed the likelihood of models representing differing distributions of circular data (hereafter, circular MLE) using the Circ_MLE package (Fitak and Johnsen, 2017) with the AICc criterion for small sample sizes. Circular data were also modelled using Bayesian probability and compared with controls (see supplementary data).

We modelled the psychometric function (Houpt and Bittner, 2018; Kirwan and Nilsson, 2019) using Stan (Carpenter et al., 2017) via the R package brms (Bürkner, 2017). We used informative priors to regularize and we inspected their influence graphically (Fig. S1). We further modelled stimulus detection at three levels of Michelson contrast (0.95, 0.1 and 0.05) treated categorically. In both cases, the model was run for 5000 sampled iterations across four chains and tested by visually inspecting chain traces, inspecting Rhat and effective sample size values, and a graphical posterior predictive check (Fig. S1).

The time taken to complete the experiment (measured from settling at the centre of the arena to the disc making contact with the wall) was recorded and compared between control, and 20–70 deg stimuli using a Kruskal–Wallis test, followed by a Mann–Whitney *U-*test, for multiple pairwise comparison of levels.

To confirm that terminal bearings were an accurate representation of animals’ orientation behaviour, available videos of experiments using 20 deg, 70 deg and control stimuli (*N*=89) were reviewed and the animals’ path tracked in full using DORIS object tracking software (https://github.com/olivierfriard/DORIS). Animals were tracked using a coordinate system with the origin at the centre of the arena, and scaled to real size using the arena diameter. The black centre of the stimulus (in the 20 and 70 deg experiments) was tracked to record its relative position to the animal. Coordinates were converted to trajectories and analysed using the Trajr package (McLean and Skowron Volponi, 2018) in R (https://www.r-project.org/). To compare initial direction of movement to terminal direction, a circle of 10 cm diameter was superimposed onto animal tracks, centred on the animal's starting position. Their initial heading was measured at the point the animal crossed the perimeter of this circle, and this was subtracted from their terminal bearing to evaluate change of direction during the trial. To assess tortuosity, trajectories were rediscretised to a constant step length: mean step length across all trajectories was 0.0038±0.0007; in order to capture all observed steps but lose minimal detail, a step size of 0.1 was chosen by visual comparison of step sizes 0.1, 1, 2 and 10. Characterising the trajectories as directed paths, the straightness of each trajectory was assessed using *r*, the length of the mean vector of turning angles (Batschelet, 1981). In case the animals’ movement was better represented as a random walk (although this did not appear to be the case from plotted trajectories), we also calculated sinuosity, a function of the mean cosine of turning angles (Benhamou, 2004). Straightness indices were compared between treatments using a Kruskal–Wallis test, followed by a Mann–Whitney *U-*test, for multiple pairwise comparison of levels, due to non-homogeneity of variances. Sinuosity was compared between treatments (control, 20 deg and 70 deg stimuli) using ANOVA, followed by Tukey's test for multiple pairwise comparison of levels.

### Loom and overhead pass stimuli

**Fig. 2.**
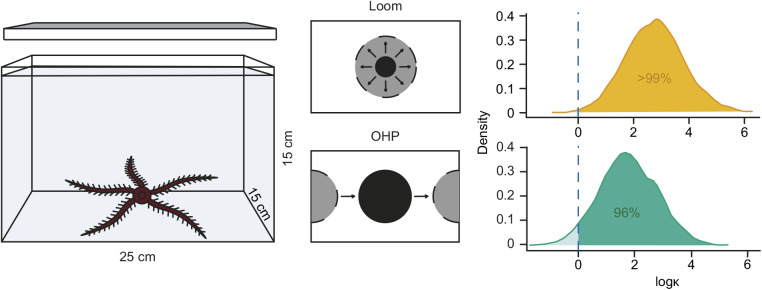
***Ophiomastix wendtii* reacts to shadows looming and passing overhead.** Animals were placed within a small tank with an overhead screen that presented a loom stimulus (a growing black circle) or an overhead pass (OHP) stimulus (a circle of the same maximum width passing across the screen). Observers were blind to the presence or absence of stimuli, and reported presence or absence of a response from filmed experiments. Curves represent modelled responses to loom and OHP stimuli, accounting for animal and observer identity. The vertical line at log κ=0 represents responses to a control stimulus. The proportion of the area under each curve that exceeds the control can be interpreted as the probability that animals responded to the stimulus.

Fourteen animals were individually placed in a small tank (15 cm×25 cm×15 cm; Fig. 2) covered on three sides with white paper backed by lightproof matte black plastic. A WLED-backlit display was positioned directly above the tank to present stimuli. We waited 4 min for the animal to habituate below a plain white screen, whereupon we presented a looming stimulus (black circle of initial diameter 13 mm/10 deg and final diameter 93 mm/70 deg, approaching over 0.5 s). In other taxa, loom responses are rapid and often mediated by dedicated neurons or reflex responses (De Franceschi et al., 2016; Oliva et al., 2007; Schlotterer, 1977; Yamawaki and Toh, 2009; Yilmaz and Meister, 2013). Cobb and Hendler (1990) recorded spiking in the radial nerve cord in response to overhead illumination and shading less than 0.1 s after stimulation in *O. wendtii*, indicating that temporal frequency is higher than that reported in sea stars (Garm and Nilsson, 2014). The main predators of *O. wendtii* are *Halichoeres* spp. and *Sparisoma* spp. (Labridae and Scaridae; Hendler, 1984). Mean observed speeds of labrids in the field range from 20 to 55 cm s^−1^ (Fulton et al., 2005; Wainwright et al., 2002), with burst and attacking speeds likely to be substantially faster (e.g. see Walker and Westneat, 2002), and parrotfish can reach more than 90 cm s^−1^ (Korsmeyer et al., 2002). A loom over 0.5 s was thus chosen to reflect ecologically relevant speeds while spanning roughly ten times the reported temporal frequency. We then waited a further 4 min before presenting a circle of constant size moving horizontally across the screen (diameter 93 mm, moving at 6 cm s^−1^). The speed of the overhead pass stimulus reflected an angular speed of 33.4 deg s^−1^ as viewed from the bottom of the tank, corresponding to *Halichoeres* passing at a distance of 30–60 cm from the viewer (20–40 cm s^−1^; Wainwright et al., 2002) or *Sparimosa* at a distance of 60–120 cm (40–80 cm s^−1^; Korsmeyer et al., 2002). Both stimuli occupied a maximum angular width of 70 deg as viewed from the bottom of the tank, which is larger than dark static stimuli demonstrated to provoke orientation responses (50 deg; Sumner-Rooney et al., 2020).

### Analysis

Animal responses were scored blind by five independent observers, using videos of the experiments with the stimulus cropped out. Observers were shown a collection of training videos demonstrating responses and non-responses, before scoring the presence of responses from 36 videos. Of these, 14 contained an overhead pass, 14 contained a loom, and eight lacked stimuli (i.e. a blank white screen) as a negative control. Timings of responses were not reported by observers, only presence or absence. The presence or absence of a recorded response was modelled using Stan (Carpenter et al., 2017) via the R package brms (Bürkner, 2017), with stimulus type as a fixed effect and animal identity and observer identity as random factors. The model was run for 4000 sampled iterations across four chains. Modelled data for loom and overhead pass stimuli were then contrasted against control data to assess the probability that animals responded to stimuli. The proportion of the response distributions greater than the control (zero) can be interpreted as the probability that the corresponding stimulus elicited a response.

## RESULTS

### Orientation experiments

Analysis of absolute bearings, prior to correction for the stimulus position, returned ΔAICc=0 for the uniform M1 distribution. No other distributions were supported with ΔAICc<2. All subsequent results are reported as bearings relative to the centre of the stimulus. Mean (±s.d.) solar irradiance during orientation experiments examining stimulus size was 379.25±291.25 W m^−2^. During experiments examining stimulus contrast, mean irradiance was 443.6±267.8 W m^−2^. For a summary of irradiances for each stimulus, see supplementary data in Table S1.

### Stimulus size

**Fig. 3.**
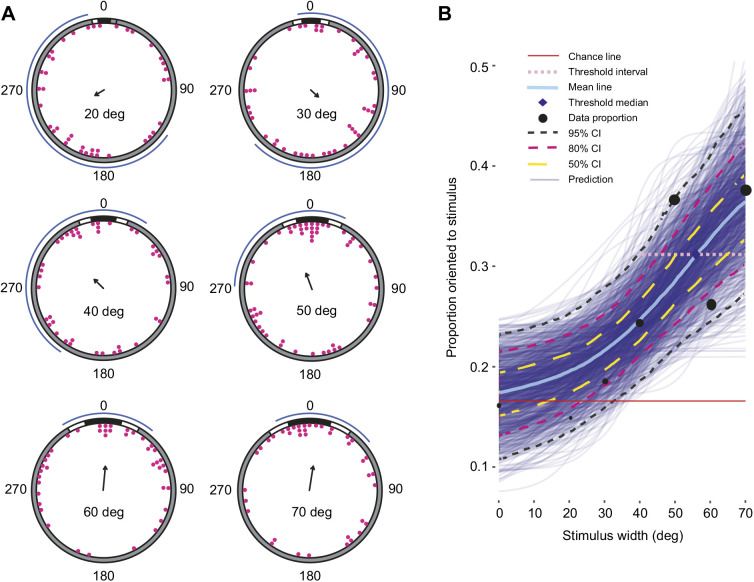
***Ophiomastix wendtii* responds to vertical bar stimuli of angular width 50** **deg** **and above.** The total angular width of the stimulus was increased from 20 to 70 deg, in 10 deg increments. Michelson contrast was 0.95. (A) Terminal bearings were disoriented relative to stimuli of 20–40 deg angular width, but clustered around the centre of the stimuli 50 deg and above. Pink dots represent one individual, presented with the stimulus once. Arrows indicate the direction and length of the mean vector; curved blue lines indicate maximum likelihood-based confidence intervals. (B) Marginal effects at the mean of orientation towards the stimulus with respect to stimulus angular width. The thin blue lines represent individual predictions. The dashed curved lines represent credible intervals (CI) for 50, 80 and 95% probability; 20 deg, *N*=40; 30 deg, *N*=43; 40 deg, *N*=41; 50 deg, *N*=52; 60 deg, *N*=42; 70 deg, *N*=40.

Animals oriented towards stimuli that occupied 50 deg of the arena wall and above, according to maximum likelihood comparisons (Fig. 3A; Table 1). Maximum likelihood comparisons returned the strongest support (AICc=0) for axial bimodal (50 deg stimulus) and unimodal (60 and 70 deg stimuli) distributions, with all circular means within 10 deg of the centre of the stimulus (Table 1). Animals presented with stimuli of 20–40 deg angular width were found to be disoriented relative to stimulus position (Fig. 3A; Table 1; AICc=0 for M1 uniform distribution).

**Table 1.**
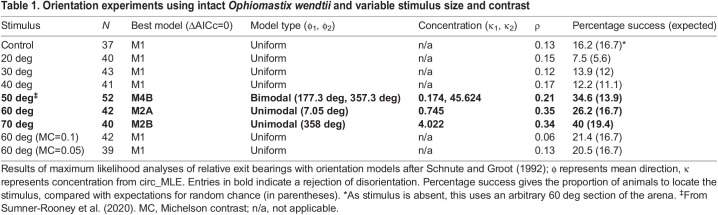
Orientation experiments using intact *Ophiomastix wendtii* and variable stimulus size and contrast

Psychometric models of success/failure to locate the stimulus were uncertain regarding a threshold point for successful orientation (Fig. 3B), but a 50% confidence interval for the threshold was estimated at 41–63 deg.

We did not observe pronounced orientation towards the internal edges of the stimulus patterns, the area of the highest local contrast, despite potential indications of this in Sumner-Rooney et al. (2020: fig. 1). We interpreted bearings as absolute deviations from 0 deg, i.e. from 0 to 180 deg, to examine this possibility, but animals did not exhibit a particular attraction to the internal stimulus edge (mean bearings 64–96.5 deg; Fig. S2).

**Fig. 4.**
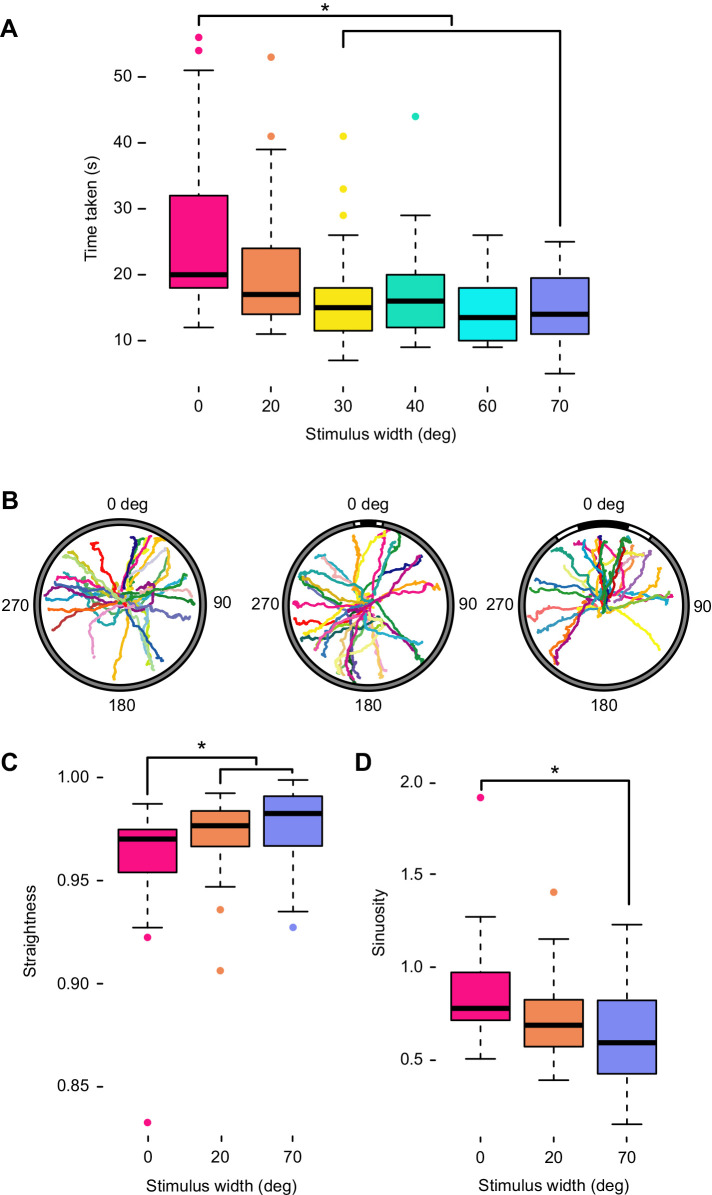
**Effects of stimulus width on speed and tortuosity.** (A) Stimulus size affected the duration of the experiments, with animals presented with a control stimulus (angular width=0 deg; *N*=37) taking significantly longer to complete trials than animals presented with 30–70 deg visual stimuli (Mann–Whitney *U-*test with Bonferroni correction for pairwise comparisons). (B) Full tracked paths of animals presented with control (left), 20 deg (centre) and 70 deg (right) stimuli. Note that not all experiments were available on video for tracking. (C) Straightness of animal paths (the length of the mean vector of turning angles, used to measure the efficiency of a directed walk) was consistently above 0.95 for all experiments, but was significantly higher in experiments using a visual stimulus than a control stimulus (Mann–Whitney *U-*test with Bonferroni correction for pairwise comparisons). (D) Sinuosity (a function of the mean cosine of turning angles, used to measure tortuosity in random walks) was significantly lower in experiments using a 70 deg than a control stimulus (Tukey's test with Bonferroni correction for pairwise comparisons). *Statistically significant differences (*P*_adj_<0.05).

Stimulus size (control, 20–70 deg) also explained a significant part of the variation in time taken to complete the experiment (Kruskal–Wallis test, χ^2^=32.219, *P*<0.0001; Fig. 4A). *Post hoc* pairwise comparisons indicated that animals presented with control stimuli took significantly longer to complete the experiment than those presented with 30–70 deg stimuli (pairwise Mann–Whitney *U-*test, Bonferroni correction for multiple comparisons, *P*_adj_<0.01), and that animals presented with the 20 deg stimulus were slower than those presented with the 60 deg stimulus (*P*_adj_=0.03).

Plotted trajectories for animals presented with control, 20 deg and 70 deg stimuli demonstrated that terminal bearing reliably represents the direction of travel (Fig. 4B). Trajectories appear to represent directed paths, and straightness indices were very high for all stimuli (0.96–0.976; Fig. 4C; Table 2). Comparisons of initial headings and terminal bearings demonstrated that animals rarely changed direction during the experiment; differences between these clustered around 0 deg (Fig. S3). Mean change in direction ranged from 358 deg (control, ρ=0.85) to 3 deg (70 deg stimulus, ρ=0.97), and maximum likelihood comparisons for all three stimuli returned strongest support for unimodal distributions around these.

**Table 2.**
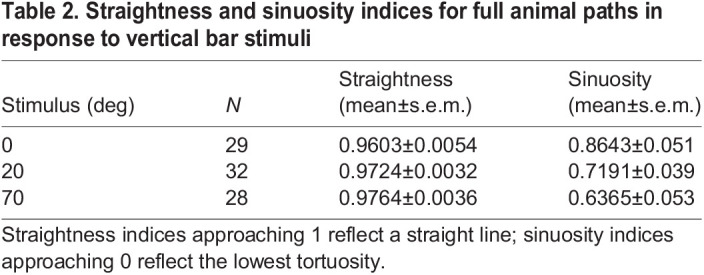
Straightness and sinuosity indices for full animal paths in response to vertical bar stimuli

While straightness was high for tracked animals presented with control, 20 deg and 70 deg stimuli, we found that it varied between them (Kruskal–Wallis test, χ^2^=11.237, *P*=0.0036), with *post hoc* analysis indicating that animals presented with visual stimuli took straighter paths than those presented with the control (pairwise Mann–Whitney *U-*test, Bonferroni correction for multiple comparisons; control versus 20 deg, *P*_adj_=0.036; control versus 70 deg, *P*_adj_=0.0062; Fig. 4C). Sinuosity also varied significantly according to stimulus size (ANOVA, *F*=9.418, *P*=0.0029), being significantly higher in animals presented with the control than the 70 deg stimulus (Tukey's multiple comparison test, *P*_adj_=0.00375; Fig. 4D).

### Contrast sensitivity

**Fig. 5.**
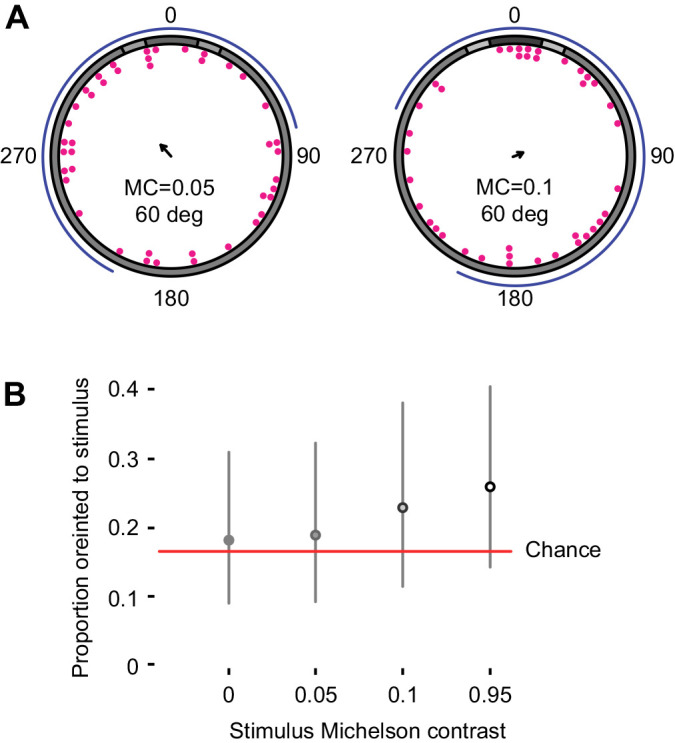
***Ophiomastix wendtii* does not respond to low-contrast vertical bar stimuli.** A vertical bar stimulus of 60 deg angular width was also presented with Michelson contrasts (MC) of 0.05 (*N*=39) and 0.1 (*N*=42). (A) Terminal bearings were disoriented relative to the centre of the stimulus. Arrows indicate the direction and length of the mean vector; curved blue lines indicate maximum likelihood-based confidence intervals. (B) Marginal effects at the mean of MC as a categorical variable on rates of successful orientation for a control stimulus (MC=0, *N*=37) and three experimental stimuli (MC of 0.05, 0.1 and 0.95). A circle represents a point estimate of the mean for each contrast level and a vertical line encloses the range of success proportions wherein the mean has 0.95 probability of falling.

Animals presented with 60 deg stimuli with Michelson contrasts of 0.1 or 0.05 were found to be disoriented by maximum likelihood analyses (Fig. 5A; Table 1). Animals were found to orient to a 60 deg stimulus with Michelson contrast of 0.95 as described above.

A categorical model of contrast treatments using Bayesian inference supported a response only to a Michelson contrast of 0.95 (Fig. 5B).

### Arm loss

**Fig. 6.**
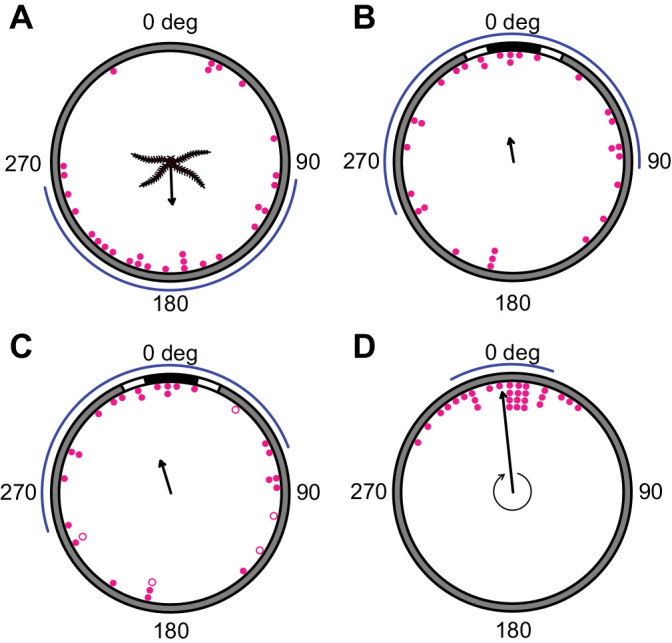
**Orientation to a vertical bar stimulus is impaired in *Ophiomastix wendtii* missing a single arm.** (A) Terminal bearings relative to the initial orientation of the missing arm demonstrate that animals tend to move directly away from their missing arm. (B) Individuals missing one arm did not orient to the centre of a stimulus of 60 deg angular width and Michelson contrast of 0.95 when placed in the arena at a random orientation. (C) When individuals that began these experiments with the missing arm facing the stimulus were removed (open circles), the remaining individuals clustered around the stimulus. (D) Comparisons of initial and terminal body orientation showed that the position of the missing arm relative to the arena did not change during experiments, i.e. animals did not turn. Arrows indicate the direction and length of the mean vector; curved blue lines indicate confidence intervals around these; *N*=29 animals.

The direction of animal movement during these experiments appears to have been affected by the orientation of the missing arm. The direction of animal movement from their initial to their terminal position in the arena, relative to the position of the missing arm, was non-uniformly distributed; animals appeared to move with the missing arm trailing (Fig. 6A; Table 3; µ=178 deg; ΔAICc=0 for M2B, unimodal clustering, ϕ=191 deg; ΔAICc<2 for M2A, unimodal clustering, ϕ=178 deg).

**Table 3.**
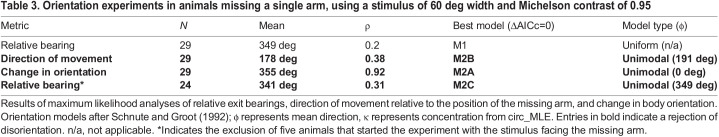
**Orientation experiments in animals missing a single arm, using a stimulus of 60** **deg** **width and Michelson contrast of 0.95**

Despite orientation to the same stimulus in intact animals, terminal bearings relative to the position of the stimulus were disoriented for animals missing one arm (Fig. 6B; Table 3; ΔAICc=0 for M1, uniform distribution, no other model returned ΔAICc<2). Five of these animals began the experiment with the armless side directed towards the stimulus (i.e. the offset between the two midpoints is below 36 deg). Given that the position of the missing arm appears to directly affect terminal bearing in an axial fashion, these animals might be expected to move away from the stimulus artifactually (four of them did so). When these five animals were excluded from analyses of orientation, terminal bearings of the remaining animals clustered around the position of the stimulus (Fig. 6C; Table 3; µ=341 deg). Maximum likelihood analyses returned strongest support for a unimodal distribution centred on the stimulus (M2C, ϕ=349 deg; Table 3), as well as additional support (ΔAICc<2) for two other unimodal distributions (ϕ=341 deg) and the M1 uniform distribution.

Animals did not substantially change their body orientation during experiments. Animals led with the same arm or arms throughout the experiment in all cases, and the difference between initial and terminal orientation of the missing arm clustered around 0 deg (Fig. 6D; Table 3; ΔAICc=0 for M2A, unimodal clustering, ϕ=356 deg, no other model returned ΔAICc<2). This accords with previous work demonstrating that brittle stars seldom change direction by rotating the central disc (Astley, 2012).

### Loom and overhead pass stimuli

Responses to dynamic stimuli were generally subtle, constituting sudden jerks of the arms or raising of the dorsal-most spines. Overall, a response was recorded for 67% of looms, 50% of overhead passes and 35% of controls. The model found that animals respond to both loom and overhead pass stimuli but not to a negative control (Fig. 2).

## DISCUSSION

Overall, our results lend further support to predator evasion being the primary role of the visual system in *O. wendtii*. Animals orient towards large, high-contrast vertical bar stimuli and exhibit potentially defensive responses to overhead shadows. Orientation is preserved in animals that have undergone natural autotomy of an arm, provided that the missing arm is not directly facing the stimulus. Arm loss is very common in ophiuroids, and Sides (1987) reported that 29.5% of collected *O*. *wendtii* were regenerating at least one arm, so the persistence of orientation behaviour despite an amputation will be relevant to a decent proportion of individuals.

### Behavioural thresholds

A distinction between behavioural responses to 40 and 50 deg stimuli emerges from our data, potentially reflecting an important threshold in the visual ecology of *O. wendtii.* Maximum likelihood analyses indicate a behavioural threshold between 40 and 50 deg angular width, within the range identified by the psychometric function, and Bayesian probability estimates indicated that animals were very likely oriented to 50 and 60 deg stimuli (see supplementary data, Table S1, Fig. S4). Although animals presented with 50, 60 and 70 deg stimuli returned the greatest support for different orientation models, we consider them all to indicate orientation towards the stimulus. The axial bimodal (M4B) distribution, recovered for the 50 deg stimulus, describes animals moving in two axially opposed directions (ϕ_1_, ϕ_2_; here, directly towards and away from the centre of the stimulus; see Table 1). However, the concentration of animals around these two directions (κ_1_, κ_2_) and their distribution between them (λ) is unequal. In this case, concentration around the stimulus was more than two orders of magnitude greater than concentration away from the stimulus (Table 1), and 73% of animals were considered by the model to be oriented towards the stimulus (λ=0.73; see Schnute and Groot, 1992). The two unimodal distributions (M2A and M2B) differ in that λ=1 for M2A; i.e. all animals are considered to be oriented in the modal direction. This is not the case in M2B (or M2C, recovered for animals missing an arm), wherein a random variable causes departure from λ=1 (Schnute and Groot, 1992). Although estimation of resolution via the psychometric function was uncertain, this analysis considers a threshold of 41–63 deg most probable (50% credible interval). This method is preferred as it incorporates relevant prior knowledge, uses responses discretised based on orientation to the target, which is more directly relevant than angular headings, and illustrates the uncertainty of the estimate.

There are several possible explanations for the observed behavioural threshold between 40 and 50 deg stimuli. First, this could reflect a limit of angular detection, i.e. directly related to the resolution of the visual system. Several morphological characteristics of a visual system can affect its performance; however, the distributed nature of the system in *Ophiomastix* means that functional interpretations based on our understanding of discrete lensed or compound eyes should be treated cautiously.

When compared with a sine grating with a period of the stimulus angular width, the centred vertical bar stimulus is isoluminant but slightly more conspicuous (greater remaining contrast) when viewed through an equivalent system with equal resolution (Fig. S5). Resolving a grating with a period of 50 deg requires a photoreceptor acceptance angle (Δ**ρ**) of 25 deg or smaller (Land, 1997; Land and Nilsson, 2012). The acceptance angle of light-adapted photoreceptors in *O. wendtii* was estimated to be 31 deg based on morphological models (Sumner-Rooney et al., 2020), a reasonable match to orientation behaviour. To estimate acceptance angles, Sumner-Rooney et al. (2020) measured aperture widths from reconstructed models of photoreceptors situated within the skeleton and surrounded by pigment granules. Photoreceptors are located within skeletal pores and bear a distal projection that expands at the tip. Apertures were measured from the base of these expansions, which are strongly reactive to antibodies raised against sea urchin r-opsin and are presumed to be the primary sensory surface. From angular apertures, photoreceptor acceptance angles were approximated on the assumption that the angular sensitivity function is Gaussian and that 99% of detected light enters through the aperture. Nilsson (2013) calculated the maximum detection angle that could reliably support low-resolution vision as 40 deg.

The composition of the photoreceptor array also contributes to the performance of the system by determining the sampling resolution. In many contiguous retinas, the inter-receptor and receptor acceptance angles are closely related, but the photoreceptors in *O. wendtii* are dispersed across the body surface and located in skeletal pores 30–40 µm apart. Despite this large translational separation, their angular separation appears to be relatively small, owing to the shallow gradient of the arm plates and the number of receptors: the mean (±s.d.) angular separation of adjacent pores (each containing a single photoreceptor), measured from synchrotron scans of *O. wendtii* (see Sumner-Rooney et al., 2018), was 6.7±2.1 deg (*N*=31) on the dorsal arm plates. This gives Δ**ρ**/Δɸ=4.67, reflecting dramatic oversampling (where Δ**ρ**/Δɸ>2; Land, 1997). The space sampled by adjacent photoreceptors in *O. wendtii* therefore overlaps considerably: in an idealised regular hexagonal array, this would result in any part of the visual scene being sampled by 12–13 photoreceptors, potentially contributing to increased sensitivity. However, we do not know whether information from the many thousands of cells is combined to form one or more images; it may be that the distribution of photoreceptors in *O. wendtii* does not represent the organisation of a retina and thus we should make such comparisons cautiously.

Although information on signal integration between adjacent photoreceptors is lacking, previous immunohistochemical studies found that afferent axons from groups of adjacent photoreceptors coalesced into bundles within the stereom. This could indicate summation or similar, but this remains purely speculative (Sumner-Rooney et al., 2018, 2020). Additionally, although individual dorsal arm plates are a relatively simple surface, these photoreceptors are found on the lateral and ventral plates, almost the full length of the arms (Sumner-Rooney et al., 2018, 2020). This would result in a highly irregularly shaped field of view, and one that would change dramatically and rapidly as the animal moved.

The behavioural threshold identified at 40–50 deg may instead under-estimate resolving power but reflect ecological relevance. Animals may be uninterested in smaller stimuli if, for example, they represent objects too distant or too small to act as effective shelters. Indeed, Sides and Woodley (1985) suggested that selection of larger shelters could contribute to niche separation between sympatric ophiocomid species. Although the occupation of a crevice is likely to be based on body size, this could confer an advantage to selectively orienting to large objects. Animals completed experiments significantly faster when presented with a high-contrast stimulus of any angular width than when presented with a control, potentially indicating that stimuli of 20–40 deg are detectable, but perhaps do not indicate a suitable shelter. We did not observe significant orientation away from these, so it is more likely that they are uninteresting than repellent, but their presence could have another impact, such as initiating movement or helping to maintain a straight path. Although the straightness of animal paths in response to a 20 deg stimulus was not found to differ statistically from either a control stimulus (*P*_adj_=0.1) or a 70 deg stimulus (*P*_adj_=0.77), there is a consistent upward trend between straightness and stimulus size (Fig. 4C) that could further support this hypothesis.

Given the strong photonegative behaviour of *O. wendtii*, we expected to find evidence for high-contrast sensitivity. However, none of our analyses supported orientation to stimuli of Michelson contrast 0.1 or 0.05. The Land optical sensitivity equation, solved for an individual photoreceptor, gives a predicted sensitivity (*S*) of 0.175 in *O. wendtii* (for broad-spectrum light given the shallow habitat; Eqn 1) (Kirschfeld, 1974; Land, 1981; Warrant and Nilsson, 1998). Here, *D*, ρ and *l* are receptor diameter (5 µm; Sumner-Rooney et al., 2018), acceptance angle (31 deg, 0.54 radians; Sumner-Rooney et al., 2020) and length (15 µm, length of observed distal expansions in Sumner-Rooney et al., 2018), respectively, and *k* is a typical absorption coefficient for a rhabdomeric photoreceptor (0.0067 µm^−1^; Warrant and Nilsson, 1998):(2)
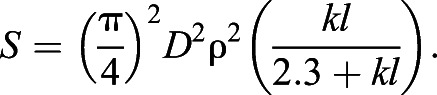
The resulting value is low, being broadly comparable to the marine worm *Vanadis* or the honeybee *Apis mellifera* (Cronin et al., 2014; Land, 1981); however, these systems both have much smaller acceptance angles of <2 deg. The very low calculated optical sensitivity predicted in *Ophiomastix* results from the dispersed nature of the system: a combination of a very small aperture (for each individual photoreceptor, rather than the pupil of an eye) and lack of focusing optics, despite the large acceptance angle. Thus, although the visual scene in the experimental arena was bright, poor optical sensitivity could somewhat limit the detection of the very low-contrast stimuli used in our experiments. The reef habitats of *O. wendtii* are shallow, bright and structurally complex, so even poor contrast sensitivity may be sufficient to detect large objects during the day, when putative shelter-seeking would be most important. However, given that the range of untested stimulus contrasts, from 0.1 to 0.95, encompasses a very wide range of natural values, future attempts to identify contrast thresholds should use intermediate levels.

### Arm loss and integration

The possibility that animals integrate photoreceptor signals across the body in a way at all analogous to facets of a compound eye seems remote; the irregularity and flexibility of the body surface would surely be a computational nightmare. Sumner-Rooney et al. (2020) noted that *O. wendtii* frequently moved by reverse rowing, with two outstretched arms leading the animal (Astley, 2012) and suggested that this indicates signal integration between adjacent arms that facilitated detection or orientation. Although we cannot conclude whether signals are compared between whole arms, we find that the removal of a single arm affects preferred direction of travel and impairs motivation and/or orientation to stimuli. The rate of reverse rowing in these experiments was around 50%, in line with previous observations of intact animals (but higher than previously observed in *Ophiocoma echinata*; Astley, 2012; Sumner-Rooney et al., 2020).

We attempted to test whether orientation ability is affected in animals that had lost an arm, but the loss itself appears to impact preferential direction of travel, with a notable trend of animals trailing the missing arm (Fig. 5A). This contradicts findings by Yoshida and Mori (unpublished data, in Yoshida, 1966) that the positively phototactic sea urchin, *Temnopleurus toreumaticus*, preferentially moves towards its operated side when a single radial nerve is cut, in the absence of a photostimulus. *Asterias amurensis* was also found to move towards arms with ocelli removed, or towards the direction of a single amputated arm (Yoshida and Ohtsuki, 1968). They suggested that this supported an inhibitory role of the ocellus, which was removed by amputation. In contrast, Clark et al. (2018) reported three modes of movement in *Ophiarachna incrassata* when one arm is isolated by cuts to the central nerve ring, two of which involved the isolated arm trailing (Clark et al., 2018) (Fig. 6), aligning with our findings. This may reflect the rather different biomechanics of ophiuroids, which use the articulated arms rather than the tube feet for locomotion and thus may be more physically impaired by such operations. It is also worth noting that both *Temnopleurus* and *Asterias* are positively phototactic and move towards the operated side, whereas both *O*. *wendtii* and *O*. *incrassata* are photonegative and move away from it; in both cases, the absence of signal may replicate an ‘on’ response.

Animals that were placed in the arena with the missing arm facing the stimulus did not orient to it; however, whether this is the direct result of biomechanical damage influencing direction or an inability to detect the stimulus is unclear. When an intact arm was oriented to the stimulus at the beginning of a trial, animals were able to locate it, indicating that detection does still play a role in the context of damage, and the biomechanical effects of arm loss do not completely override this behaviour. Similarly, Yoshida and Kobayashi (unpublished data, in Yoshida, 1966) found that *T. toreumaticus* never moved directly towards a light source on their operated side, but they did observe animals rotate and then lead with an intact side. Drawing conclusions about integration from these data would be premature, but it is notable that even where a missing arm faces the centre of a stimulus of 60 deg angular width, the stimulus is likely to be partially within the field of view of the two adjacent, intact arms. If so, detection by single or non-adjacent arms would not appear to be sufficient to affect orientation. The nerve ring is intact, so communication between the remaining arms (as observed by Stubbs, 1983) should be unimpeded.

To clarify whether two adjacent arms are required for stimulus detection, future experiments could use animals with two arms removed, either side of a single intact arm, and place animals in the arena with this intact arm facing the stimulus. If one arm is sufficient for detection, animals might be expected to orient towards the stimulus, but if communication between adjacent arms is required, we would expect disorientation. If the removal of two arms has a greater impact on direction of travel than the presence of the stimulus, animals may orient directly away from the stimulus, assuming that the animal will move away from removed arms as observed in the current study (and in line with similar experiments in *Asterias* by Yoshida and Ohtsuki, 1968). If the latter is not the case, then orienting animals with the pair of intact, adjacent arms facing the stimulus may recover orientation behaviour. This potentially has important ramifications for animal survival; in predation experiments, Hendler (1984) exposed 12 specimens of *O*. *wendtii* on an open reef floor, alongside individuals of three other species, and reported 27 attacks on four of them in the space of 3 min.

### Dynamic stimuli

Our model indicates that animals were highly likely to respond to both loom and overhead pass stimuli, compared with control experiments. Looms are ecologically relevant as they may represent an approaching threat, and loom responses are widespread in other taxa (e.g. Oliva et al., 2007; Yamawaki and Toh, 2009; Yilmaz and Meister, 2013). Uniform shading or shadows passing overhead can produce mixed or different responses in other taxa, such as freezing, probably signifying a reduced level of immediate threat (De Franceschi et al., 2016). However, distinguishing between these threats requires more sophisticated processing than we find in *O. wendtii*. We tried to approximate ecologically and physiologically relevant speeds and sizes in our experiments, based on existing knowledge of both *O*. *wendtii* and its predators, that might be most likely to evoke responses.

However, we cannot conclude that *Ophiomastix* responds specifically to dynamic stimuli, as they may have responded to the appearance of a dark object overhead. Response time, which could indicate a critical angular size of an approaching object, was not recorded. Nonetheless, responsiveness to the appearance of a dark stimulus demonstrates a second function of the visual system in *O. wendtii* in addition to shelter-seeking. This may reflect the high predation pressure in the crowded reef rubble habitats of *O. wendtii*, with fish readily attacking exposed animals (Hendler, 1984; L.S.-R., personal observation). Responses to dynamic or appearing stimuli do not require spatial resolution, and ‘alarm’ photoreception has been described as a distinct class of visual task by Nilsson (2013). All examples of alarm photoreception therein (e.g. ark clams and sabellid worms) are taxa with distributed visual systems, where individual units may not provide image information (Nilsson, 1994, 2013). The detection of shadows by *O. wendtii* fits well into this category, suggesting that this response may not be reliant on image formation. In addition, Nilsson (1994) found that both ark clams and sabellids extensively oversampled the visual field, with dozens or even hundreds of ommatidia overlapping. The sampling predicted in *O. wendtii* is less dense than these examples, but may still benefit from filtering out false alarms, reduced vulnerability to injury or biofouling, and increasing signal-to-noise ratio by pooling photoreceptor signals.

### Conclusions

*Ophiomastix wendtii* is able to detect and respond to high-contrast static and appearing stimuli, indicating uses for photoreception and vision in both habitat selection and defensive behaviours. A behavioural threshold in orientation responses to stimuli of 50 deg width and above could be indicative of either a limit of detection or motivation. Anatomical evidence indicates that photoreceptor acceptance angles would be a good match for the required resolution. We observed the extinction of orientation to 60 deg stimuli at Michelson contrasts of 0.1 and below, and calculated poor predicted optical sensitivity from morphology. We also report a response to moving stimuli for the first time in brittle stars, although its specificity cannot be confirmed at this stage. However, this indicates a defensive role for vision in addition to shelter-seeking. How signals are integrated between photoreceptors, arm segments or entire arms remains a considerable challenge. Future experiments could help clarify whether signal comparison between adjacent arms could play a role in detecting and locating visual stimuli.

## Supplementary Material

Supplementary information

## References

[JEB236653C1] Aizenberg, J. and Hendler, G. (2004). Designing efficient microlens arrays: lessons from nature. *J. Mater. Chem.* 14, 2066. 10.1039/b402558j

[JEB236653C2] Arshavskii, Y. I., Kashin, S. M., Litvinova, N. M., Orlovskii, G. N. and Feldman, A. G. (1976). Coordination of arm movement during locomotion in ophiurans. *Neurophysiology* 8, 404-410. 10.1007/BF01063603 980174

[JEB236653C3] Astley, H. C. (2012). Getting around when you're round: quantitative analysis of the locomotion of the blunt-spined brittle star, *Ophiocoma echinata*. *J. Exp. Biol.* 215, 1923-1929. 10.1242/jeb.068460 22573771

[JEB236653C4] Batschelet, E. (1981). *Circular Statistics in Biology*. London: Academic Press Inc.

[JEB236653C5] Battelle, B. A. (2016). Simple eyes, extraocular photoreceptors and opsins in the American horseshoe crab. *Integr. Comp. Biol.* 56, 809-819. 10.1093/icb/icw093 27444526

[JEB236653C6] Benhamou, S. (2004). How to reliably estimate the tortuosity of an animal's path: straightness, sinuosity, or fractal dimension? *J. Theor. Biol.* 229, 209-220. 10.1016/j.jtbi.2004.03.016 15207476

[JEB236653C7] Blevins, E. and Johnsen, S. (2004). Spatial vision in the echinoid genus *Echinometra*. *J. Exp. Biol.* 207, 4249-4253. 10.1242/jeb.01286 15531646

[JEB236653C8] Bok, M. J., Nilsson, D. E. and Garm, A. (2019). Photoresponses in the radiolar eyes of the fan worm *Acromegalomma vesiculosum*. *J. Exp. Biol.* 22. 10.1242/jeb.212779 31727758

[JEB236653C9] Bürkner, P.-C. (2017). brms: an R package for Bayesian multilevel models using Stan. *J. Stat. Softw.* 80, 1-28. 10.18637/jss.v080.i01

[JEB236653C10] Carpenter, B., Gelman, A., Hoffman, M. D., Lee, D., Goodrich, B., Betancourt, M., Brubaker, M., Guo, J., Li, P. and Riddell, A. (2017). Stan: a probabilistic programming language. *J. Stat. Software* 1. 10.18637/jss.v076.i01 PMC9788645 36568334

[JEB236653C11] Clark, E. G., Kanauchi, D., Kano, T., Aonuma, H., Briggs, D. E. G. and Ishiguro, A. (2018). The function of the ophiuroid nerve ring: how a decentralized nervous system controls coordinated locomotion. *J. Exp. Biol.* 222, jeb.192104. 10.1242/jeb.192104 30464042

[JEB236653C12] Cobb, J. L. S. and Hendler, G. (1990). Neurophysiological characterisation of the photoreceptor system in a brittlestar, *Ophiocoma wendtii* (Echinodermata: Ophiuroidea). *Comp. Biochem. Physiol. A* 97, 329-333. 10.1016/0300-9629(90)90619-4

[JEB236653C13] Cronin, T. W. and Johnsen, S. (2016). Extraocular, non-visual, and simple photoreceptors: an introduction to the symposium. *Integr. Comp. Biol.* 56, 758-763. 10.1093/icb/icw106 27493148

[JEB236653C14] Cronin, T. W., Johnsen, S., Marshall, N. J. and Warrant, E. J. (2014). *Visual Ecology*. Princeton: Princeton University Press.

[JEB236653C15] De Franceschi, G., Vivattanasarn, T., Saleem, A. B. and Solomon, S. G. (2016). Vision guides selection of freeze or flight defense strategies in mice. *Curr. Biol.* 26, 2150-2154. 10.1016/j.cub.2016.06.006 27498569

[JEB236653C16] Delroisse, J., Ullrich-Lüter, E., Ortega-Martinez, O., Dupont, S., Arnone, M.-I., Mallefet, J. and Flammang, P. (2014). High opsin diversity in a non-visual infaunal brittle star. *BMC Genomics* 15, 1035. 10.1186/1471-2164-15-1035 25429842 PMC4289182

[JEB236653C17] Fitak, R. R. and Johnsen, S. (2017). Bringing the analysis of animal orientation data full circle: model-based approaches with maximum likelihood. *J. Exp. Biol.* 76, jeb.167056. 10.1242/jeb.167056 PMC6514460 28860118

[JEB236653C18] Fulton, C. J., Bellwood, D. R. and Wainwright, P. C. (2005). Wave energy and swimming performance shape coral reef fish assemblages. *Proc. R. Soc. B Biol. Sci.* 272, 827-832. 10.1098/rspb.2004.3029 PMC1599856 15888415

[JEB236653C19] Garm, A. and Nilsson, D.-E. (2014). Visual navigation in starfish: first evidence for the use of vision and eyes in starfish. *Proc. R. Soc. London. Ser. B, Biol. Sci.* 281, 1-8. 10.1098/rspb.2013.3011 PMC3896028 24403344

[JEB236653C20] Glaser, O. C. (1907). Movement and problem-solving in *Brevispina*. *J. Exp. Zool.* 4, 203-220. 10.1002/jez.1400040203

[JEB236653C21] Hendler, G. (1984). Brittlestar color-change and phototaxis (Echinodermata: Ophiuroidea: Ophiocomidae). *Mar. Ecol.* 5, 379-401. 10.1111/j.1439-0485.1984.tb00131.x

[JEB236653C22] Houpt, J. W. and Bittner, J. L. (2018). Analyzing thresholds and efficiency with hierarchical Bayesian logistic regression. *Vision Res.* 148, 49-58. 10.1016/j.visres.2018.04.004 29678536

[JEB236653C23] Johnsen, S. (1997). Identification and localization of a possible rhodopsin in the echinoderms *Asterias forbesi* (Asteroidea) and *Ophioderma brevispinum* (Ophiuroidea). *Biol. Bull.* 193, 97-105. 10.2307/1542739 9290215

[JEB236653C24] Kingston, A. C. N. and Cronin, T. W. (2016). Diverse distributions of extraocular opsins in crustaceans, cephalopods, and fish. *Integr. Comp. Biol.* 56, 820-833. 10.1093/icb/icw022 27252200

[JEB236653C25] Kirschfeld, K. (1974). The absolute sensitivity of lens and compound eyes. *Zeitschrift für Naturforsch.* 29, 592-596. 10.1515/znc-1974-9-1023 4278301

[JEB236653C26] Kirwan, J. D. and Nilsson, D. E. (2019). A millipede compound eye mediating low-resolution vision. *Vision Res.* 165, 36-44. 10.1016/j.visres.2019.09.003 31622904

[JEB236653C27] Kirwan, J. D., Bok, M. J., Smolka, J., Foster, J. J., Hernández, J. C. and Nilsson, D.-E. (2018). The sea urchin *Diadema africanum* uses low resolution vision to find shelter and deter enemies. *J. Exp. Biol.* 221, jeb176271. 10.1242/jeb.176271 29739834

[JEB236653C28] Korsmeyer, K. E., Steffensen, J. F. and Herskin, J. (2002). Energetics of median and paired fin swimming, body and caudal fin swimming, and gait transition in parrotfish (*Scarus schlegeli*) and triggerfish (*Rhinecanthus aculeatus*). *J. Exp. Biol.* 205, 1253-1263. 11948202 10.1242/jeb.205.9.1253

[JEB236653C29] Land, M. F. (1981). Optics and vision in invertebrates. In *Handbook of Sensory Physiology: Vision in Invertebrates A: Invertebrate Photoreceptors*, Vol. VII/6B (ed. H. Autrum), pp. 471-592. Berlin: Springer.

[JEB236653C30] Land, M. F. (1997). Visual acuity in insects. *Annu. Rev. Entomol.* 42, 147-177. 10.1146/annurev.ento.42.1.147 15012311

[JEB236653C31] Land, M. F. and Nilsson, D.-E. (2012). *Animal Eyes*, 2nd edn. Oxford: Oxford University Press.

[JEB236653C32] Mao, S., Dong, E., Xu, M., Jin, H., Li, F. and Yang, J. (2013). Design and development of starfish-like robot: Soft bionic platform with multi-motion using SMA actuators. In IEEE International Conference on Robotics and Biomimetics (ROBIO), Shenzhen, pp. 91-96.

[JEB236653C33] McLean, D. J. and Skowron Volponi, M. A. (2018). trajr: an R package for characterisation of animal trajectories. *Ethology* 124, 440-448. 10.1111/eth.12739

[JEB236653C34] Nilsson, D. E. (1994). Eyes as optical alarm systems in fan worms and ark clams. *Philos. Trans. R. Soc. B Biol. Sci.* 346, 195-212. 10.1098/rstb.1994.0141

[JEB236653C35] Nilsson, D.-E. (2013). Eye evolution and its functional basis. *Vis. Neurosci.* 30, 5-20. 10.1017/S0952523813000035 23578808 PMC3632888

[JEB236653C36] O'Hara, T. D., Hugall, A. F., Cisternas, P. A., Boissin, E., Bribiesca-Contreras, G., Sellanes, J., Paulay, G. and Byrne, M. (2019). Phylogenomics, life history and morphological evolution of ophiocomid brittlestars. *Mol. Phylogenet. Evol.* 130, 67-80. 10.1016/j.ympev.2018.10.003 30308280

[JEB236653C37] Oliva, D., Medan, V. and Tomsic, D. (2007). Escape behavior and neuronal responses to looming stimuli in the crab *Chasmagnathus granulatus* (Decapoda: Grapsidae). *J. Exp. Biol.* 210, 865-880. 10.1242/jeb.02707 17297146

[JEB236653C38] Ramirez, M. D., Speiser, D. I., Pankey, S. M. and Oakley, T. H. (2011). Understanding the dermal light sense in the context of integrative photoreceptor cell biology. *Vis. Neurosci.* 28, 265-279. 10.1017/S0952523811000150 21736861

[JEB236653C39] Schlotterer, G. R. (1977). Response of the locust descending movement detector neuron to rapidly approaching and withdrawing visual stimuli. *Can. J. Zool.* 55, 1372-1376. 10.1139/z77-179

[JEB236653C40] Schnute, J. T. and Groot, K. (1992). Statistical analysis of animal orientation data. *Anim. Behav.* 43, 15-33. 10.1016/S0003-3472(05)80068-5

[JEB236653C41] Sides, E. M. (1987). An experimental study of the use of arm regeneration in estimating rates of sublethal injury on brittle-stars. *J. Exp. Mar. Bio. Ecol.* 106, 1-16. 10.1016/0022-0981(87)90144-4

[JEB236653C42] Sides, E. M. and Woodley, J. D. (1985). Niche separation in three species of *Ophiocoma* (Echinodermata: Ophiuroidea) in Jamaica, West Indies. *Bull. Mar. Sci.* 36, 701-715.

[JEB236653C43] Speiser, D. I., Eernisse, D. J. and Johnsen, S. (2011). A chiton uses aragonite lenses to form images. *Curr. Biol.* 21, 665-670. 10.1016/j.cub.2011.03.033 21497091

[JEB236653C44] Stubbs, T. R. (1983). Some aspects of the neurobiology of ophiuroids: with special reference to *Ophiura texturata* (L.) (Echinodermata, Ophiuroidea). PhD thesis, University of St Andrews.

[JEB236653C45] Sumner-Rooney, L., Kirwan, J. D., Lowe, E. K. and Ullrich-Lüter, E. (2020). Extraocular vision in a brittle star is mediated by chromatophore movement in response to ambient light. *Curr. Biol.* 30, 1-9. 10.1016/j.cub.2019.11.042 31902727

[JEB236653C46] Sumner-Rooney, L., Rahman, I. A., Sigwart, J. D. and Ullrich-Lüter, E. M. (2018). Whole-body photoreceptor networks are independent of ‘lenses’ in brittle stars. *Proc. R. Soc. B* 285, 20172590. 10.1098/rspb.2017.2590 PMC5805950 29367398

[JEB236653C47] Tong, D., Rozas, N. S., Oakley, T. H., Mitchell, J., Colley, N. J. and McFall-Ngai, M. J. (2009). Evidence for light perception in a bioluminescent organ. *Proc. Natl. Acad. Sci. USA* 106, 9836-9841. 10.1073/pnas.0904571106 19509343 PMC2700988

[JEB236653C48] Ullrich-Lüter, E. M., Dupont, S., Arboleda, E., Hausen, H. and Arnone, M. I. (2011). Unique system of photoreceptors in sea urchin tube feet. *Proc. Natl. Acad. Sci. USA* 108, 8367-8372. 10.1073/pnas.1018495108 21536888 PMC3100952

[JEB236653C49] von Salvini-Plawen, L. and Mayr, E. (1977). On the evolution of photoreceptors and eyes. *Evol. Biol.* 10, 207-263. 10.1007/978-1-4615-6953-4_4

[JEB236653C50] Wainwright, P. C., Bellwood, D. R. and Westneat, M. W. (2002). Ecomorphology of locomotion in labrid fishes. *Environ. Biol. Fishes* 65, 47-62. 10.1023/A:1019671131001

[JEB236653C51] Wakita, D., Kagaya, K. and Aonuma, H. (2020). A general model of locomotion of brittle stars with a variable number of arms. *J. R. Soc. Interface* 17, 20190374. 10.1098/rsif.2019.0374 31910773 PMC7014800

[JEB236653C52] Walker, J. A. and Westneat, M. W. (2002). Kinematics, dynamics, and energetics of rowing and flapping propulsion in fishes. *Integr. Comp. Biol.* 42, 1032-1043. 10.1093/icb/42.5.1032 21680385

[JEB236653C53] Warrant, E. J. and Nilsson, D. E. (1998). Absorption of white light in photoreceptors. *Vision Res.* 8, 195-207. 10.1016/S0042-6989(97)00151-X 9536349

[JEB236653C54] Watanabe, W., Suzuki, S., Kano, T. and Ishiguro, A. (2011). Moving right arm in the right place: ophiuroid-inspired omnidirectional robot driven by coupled dynamical systems. In IEEE/RSJ International Conference on Intelligent Robots and Systems, San Francisco, CA, pp. 1895-1900.

[JEB236653C55] Yamawaki, Y. and Toh, Y. (2009). Responses of descending neurons to looming stimuli in the praying mantis *Tenodera aridifolia*. *J. Comp. Physiol. A* 195, 253-264. 10.1007/s00359-008-0403-6 19093123

[JEB236653C56] Yang, S. and Aizenberg, J. (2005). Microlens arrays with integrated pores. *Nano Today* 8, 40-46. 10.1016/S1369-7021(05)71288-8

[JEB236653C57] Yerramilli, D. and Johnsen, S. (2010). Spatial vision in the purple sea urchin *Strongylocentrotus purpuratus* (Echinoidea). *J. Exp. Biol.* 213, 249-255. 10.1242/jeb.033159 20038658

[JEB236653C58] Yilmaz, M. and Meister, M. (2013). Rapid innate defensive responses of mice to looming visual stimuli. *Curr. Biol.* 23, 2011-2015. 10.1016/j.cub.2013.08.015 24120636 PMC3809337

[JEB236653C59] Yoshida, M. (1966). Photosensitivity. In *Physiology of Echinodermata* (ed. R. A. Boolootian), pp. 435-464. New York: John Wiley & Sons, Ltd.

[JEB236653C60] Yoshida, M. and Ohtsuki, H. (1968). The phototactic behavior of the starfish, *Asterias amurensis* Lütken. *Biol. Bull.* 134, 516-532. 10.2307/1539869

