# Supplementary material for: Run and hide: visual performance in a brittle star
Source: J Exp Biol. 2021 Jun 8;224(11):jeb236653. doi: 10.1242/jeb.236653 (PMC8214828; doi:10.1242/jeb.236653)
Supplement: Supplementary information [file jexbio-224-236653-s1.pdf]

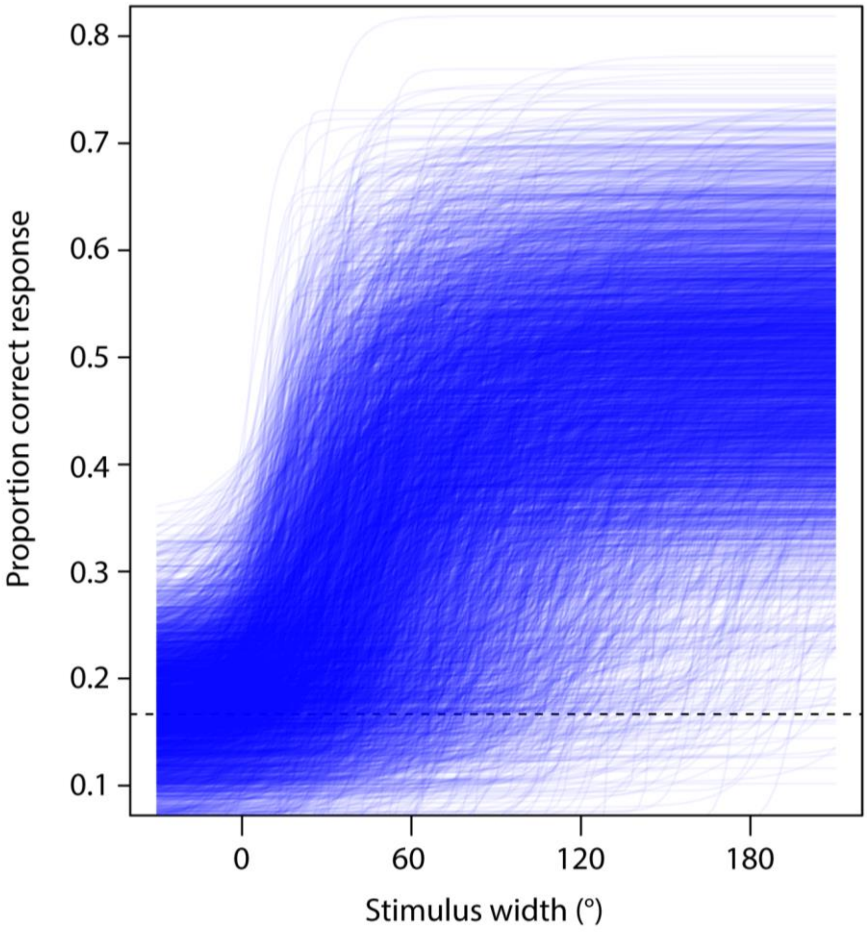

Figure S1. Prior predictive plot used for psychometric modelling of threshold stimulus width. Blue lines are 5000 possible model fits produced using the prior information only for the psychometric function model. The range and concentration of fitted lines illustrate the prior bias. The priors were informed by prior knowledge of *O. wendtii* response robustness to visual stimuli (Sumner-Rooney et al., 2020). High response proportions (> 0.7) were very unlikely, as are those well below chance. The black dashed line represents the mostly likely proportion by random chance (0.167).

Journal of Experimental Biology • Supplementary information

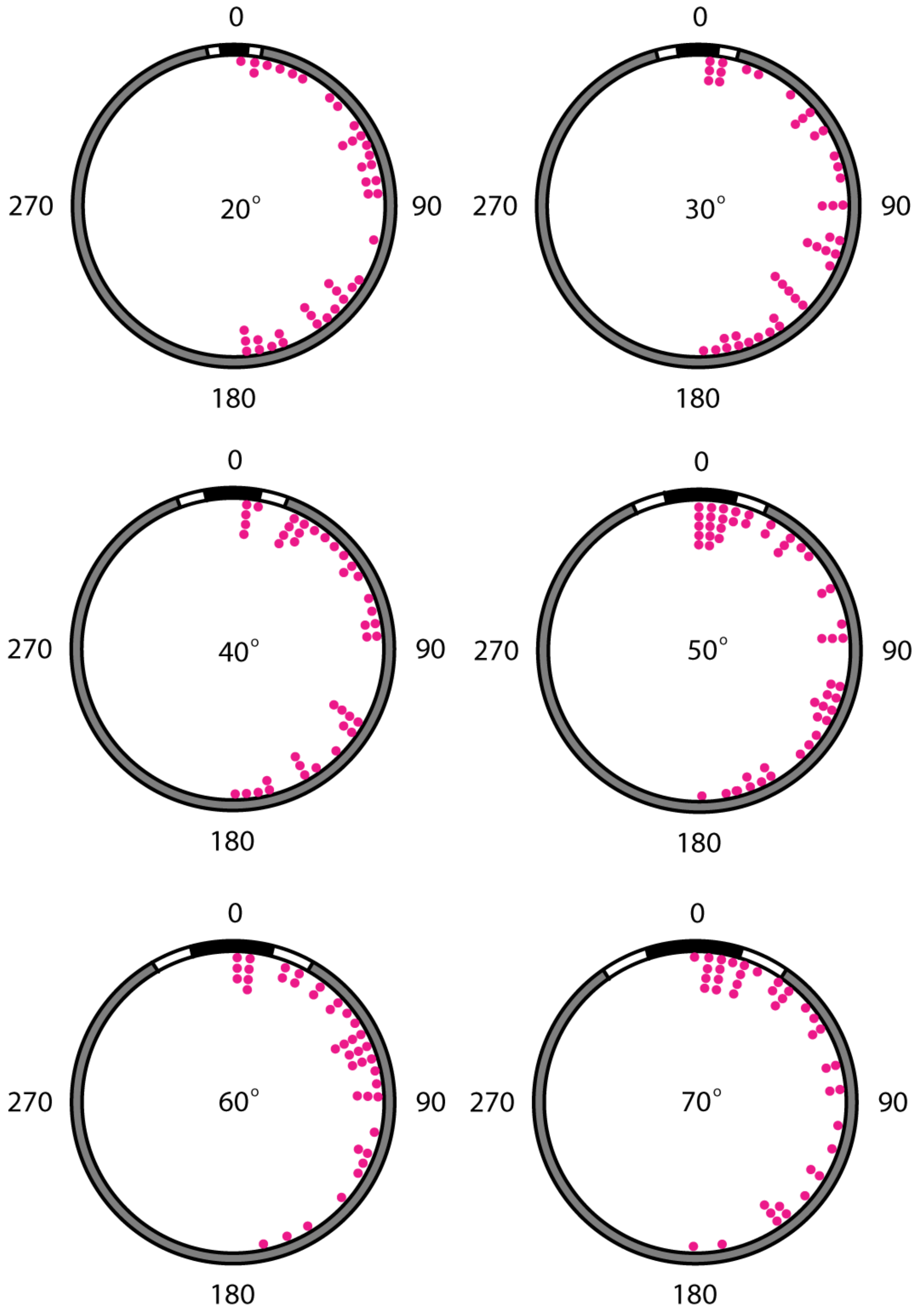

Figure S2. Orientation of *Ophiomastix wendtii* to stimuli of variable angular width, collapsed to 0-180°. Sumner-Rooney et al. (2020) proposed that animals might orient to the areas of highest local contrast, the internal black-white edge of the stimulus. Orientation data (0-360°, Figure 2) were collapsed to 0-180° to visually inspect this; animals do not appear to specifically cluster at this edge.

Journal of Experimental Biology • Supplementary information

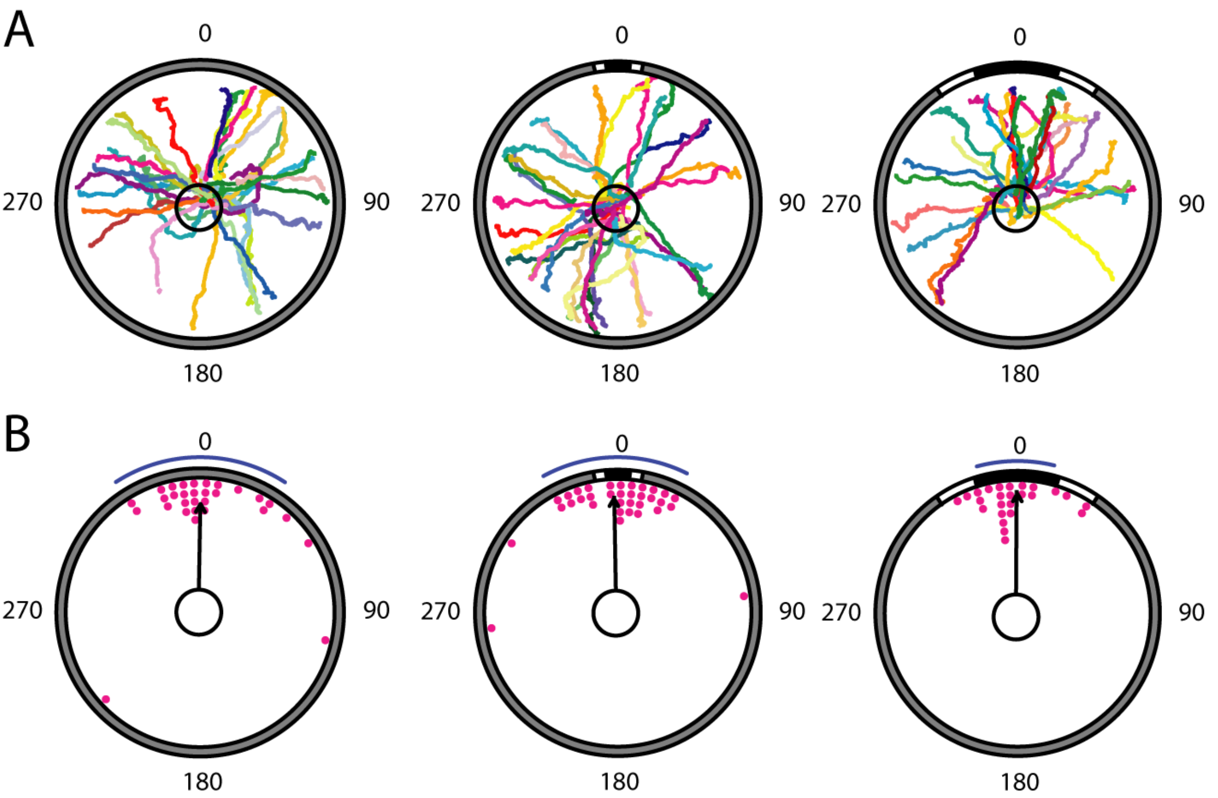

Figure S3. Differences between initial heading and terminal bearing of animals presented with control, 20°, and 70° stimuli. A. The heading of tracked animals was measured as they crossed the perimeter of a superimposed circle (diameter 10 cm) at the centre of the arena. **B**. Initial headings were subtracted from terminal bearings (see Figure 3). The differences clustered around 0° for all three stimuli, indicating little change in direction during experiments. Arrows indicate the direction and length of the mean vector; blue brackets indicate confidence intervals around these.

Journal of Experimental Biology • Supplementary information

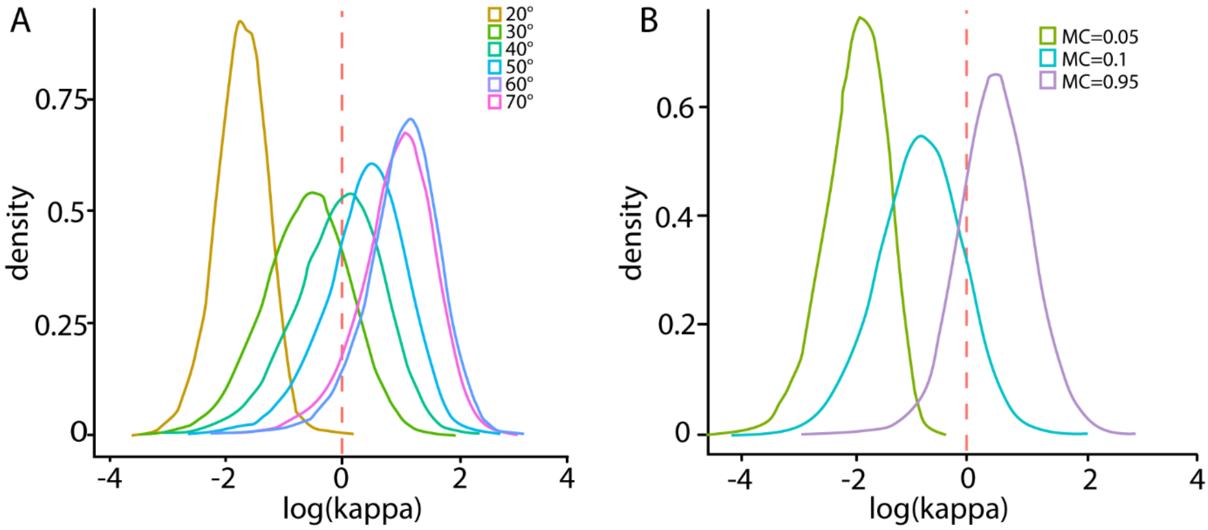

Figure S4. Comparisons of animal orientation modelled as von Mises distributions. Each curve represents modelled orientations to a single experimental stimulus. The vertical line at log(kappa)=0 represents animal orientation to a control stimulus (from Sumner-Rooney et al., 2020). The proportion of the area under each curve that overlaps with the control can be interpreted as the probability that the animals were disoriented. See Table S1.

Journal of Experimental Biology • Supplementary information

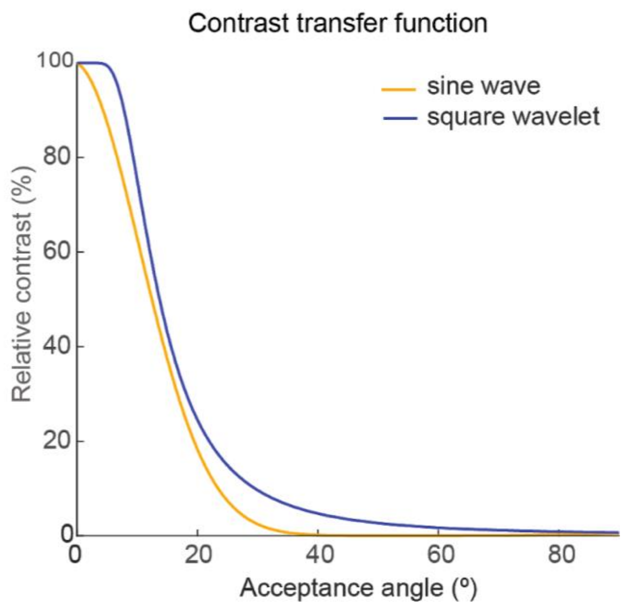

Figure S5. Contrast transfer function of square wavelet and sine signals at 30° width, showing the Michelson contrast remaining when Gaussian filtered through varying acceptance angles (full width at half maximum of the angular sensitivity function). Greater contrast remains for the square wave, making it as visible as the sine with worse visual acuity.

Table S1. Bayesian analyses of orientation and ambient light conditions during experiments. Relative headings were modelled according to Von Mises (angular) distributions. Prob. oriented indicates the probability that animals are more concentrated than would be expected by chance, according to a Bayesian model (based on statistical contrast with control data from Sumner-Rooney et al. 2020). Mean solar irradiance during orientation experiments June-July 2019 and May 2017. Compiled from mean irradiance measurements taken across a 180° view every 15 minutes from 10am-3:15pm on the days of the experiments. Data provided by the Physical Monitoring Program of the Smithsonian Tropical Research Institute. Bold indicates the rejection of disorientation by circ_MLE (main text, Table 1) for comparison.

*From Sumner-Rooney et al. (2020)

**Stimulus**	**n**	**Prob. oriented (vs. control)**	**Mean irradiance ($W m^{-2}$)**	**Standard deviation ($W m^{-2}$)**
20°	40	0.19	409.09	260.66
30°	43	0.49	337.34	173.65
40°	41	0.74	482.46	314.79
**50°***	**52**	**0.95**	**269.42**	**146.32**
**60°**	**42**	**0.92**	**598.4**	**261.49**
**70°**	**40**	**0.74**	**68.98**	**31.21**
60° (MC=0.1)	42	0.85	200.5	96.73
60° (MC=0.05)	39	0.72	686.78	125.89
